# Matricellular Regulation of Alveolar Epithelial Cell Plasticity in Neonatal Lung Injury: Insights from CCN5 and Beyond

**DOI:** 10.18103/mra.v13i10.6931

**Published:** 2025-10

**Authors:** Najla Fiaturi, Heber C Nielsen, John Castellot

**Affiliations:** 1 Department of Medical Education, Tufts University School of Medicine, Boston MA, USA; 2 Graduate Program in Drug Development, Graduate School of Biomedical Sciences, Tufts University School of Medicine, Boston MA, USA; 3 Graduate Program in Genetics, Molecular, and Cell Biology, Tufts Graduate Program in Biomedical Sciences, and Department of Pediatrics, Tufts Medical Center, Boston MA, USA

**Keywords:** CCN5, alveolar epithelial cells, bronchopulmonary dysplasia, matricellular signaling, neonatal lung injury, extracellular matrix, epithelial plasticity, regenerative biology

## Abstract

Neonatal lung injury remains a major cause of morbidity among preterm infants and is a leading antecedent of bronchopulmonary dysplasia (BPD), a condition characterized by arrested alveolar development, inflammation, and extracellular matrix (ECM) remodeling. Despite significant advances in neonatal care, the mechanisms governing lung repair and regeneration remain incompletely understood. Recent research highlights the crucial role of matricellular proteins that orchestrate cell signaling, differentiation, and tissue remodeling in modulating–epithelial responses to injury. Among these, the CCN (CYR61/CTGF/NOV) family of proteins has emerged as a pivotal regulator of epithelial mesenchymal communication, with diverse and context-dependent functions in development, fibrosis, and regeneration.

This review examines the distinct role of **CCN5 (WISP2)** within this family as a modulator of alveolar epithelial cell (AEC) plasticity in neonatal lung injury. We integrate findings from our own work with recent advances in CCN biology to elucidate how CCN5 influences epithelial survival, epithelial–mesenchymal transition (EMT), mitochondrial homeostasis, and intercellular signaling in hyperoxic injury. The review also contrasts CCN5 with other CCN members implicated in fibrotic lung diseases and situates it within broader matricellular signaling networks. By defining how CCN5 mediates epithelial resilience and tissue homeostasis, we propose it as a potential therapeutic target for mitigating BPD and enhancing regenerative outcomes in premature infants.

## 1. Introduction

The neonatal period represents a critical phase of lung morphogenesis, during which the late saccular and alveolar stages overlap with the postnatal transition to air breathing. For preterm infants born before 32 weeks of gestation, this transition occurs before full maturation of the alveolar epithelium, predisposing the immature lung to mechanical, oxidative, and inflammatory insults. These injurious stimuli—most notably oxygen therapy and mechanical ventilation—disrupt the tightly coordinated signaling required for alveolarization, leading to bronchopulmonary dysplasia (BPD), a chronic lung disease of prematurity that persists despite major improvements in neonatal care.^6,7^

BPD pathogenesis reflects a multifactorial interplay among inflammation, oxidative stress, mechanical injury, and dysregulated ECM remodeling. At the cellular level, the alveolar epithelium—particularly type II alveolar epithelial cells (AEC2s)—is central to both injury and repair. AEC2s produce pulmonary surfactant to maintain alveolar stability and act as progenitors capable of regenerating type I cells (AEC1s) after damage.^5^ Their dysfunction contributes to impaired gas exchange, fibrosis, and loss of regenerative capacity. Recent studies have shown that AEC2 behavior during repair is governed not only by intrinsic signaling (e.g., Wnt/**β**-catenin, Notch, and Hippo/YAP) but also by extracellular cues provided by the surrounding matrix and stromal cells.^10^

Among extracellular cues, matricellular proteins—a specialized class of secreted ECM-associated factors—have gained prominence for their regulatory roles in lung development and pathology. Unlike structural ECM components such as collagen or elastin, matricellular proteins fine-tune cellular responses by interacting with integrins, growth factors, and cytokines to modulate adhesion, proliferation, migration, and differentiation. The CCN family (CYR61/CCN1, CTGF/CCN2, NOV/CCN3, WISP1/CCN4, WISP2/CCN5, WISP3/CCN6) constitute a core group of these regulators. The CCN family comprises six matricellular proteins that share conserved structural domains but differ in biological function. The first three members identified—CYR61, CTGF, and NOV—are now officially designated as CCN1, CCN2, and CCN3, respectively. Subsequent discoveries added three related proteins—WISP1, WISP2, and WISP3—which correspond to CCN4, CCN5, and CCN6 in the current nomenclature.^11_13^

In the lung, CCN proteins have been implicated in fibrosis, angiogenesis, and epithelial–mesenchymal communication. CCN2 (CTGF) is well established as a profibrotic mediator, driving fibroblast activation and ECM deposition in idiopathic pulmonary fibrosis (IPF) and BPD.^8^ CCN1 (CYR61) exhibits dual roles—supporting angiogenesis and repair in some contexts while amplifying inflammation in others.^14^ In contrast, CCN5 (WISP2) is structurally distinct, lacking the cysteine-knot (CT) domain that mediates many CCN-protein interactions. This truncation confers a non-fibrogenic, epithelial-protective phenotype observed in cardiac, hepatic, and adipose models, suggesting CCN5 may counterbalance the fibrotic actions of its family members.^1_3, 8^

Despite these findings, the role of CCN5 in neonatal lung development and injury remains poorly understood. Preliminary evidence from our laboratory and others indicates that CCN5 supports epithelial integrity, preserves mitochondrial function, and suppresses EMT during hyperoxic injury.^1_3, 20^ Together, these findings position CCN5 as a potential regulatory hub at the intersection of epithelial resilience, ECM remodeling, and redox homeostasis.

## 2. Lung Development and the Pathophysiology of Bronchopulmonary Dysplasia (BPD)

Lung development follows five highly regulated stages: embryonic, pseudoglandular, canalicular, saccular, and alveolar.^6^ The transition from the saccular to the alveolar phase occurs late in gestation and extends into early childhood, meaning preterm birth interrupts a phase of morphogenesis essential for efficient gas exchange.

During normal development, epithelial, endothelial, and mesenchymal cells coordinate through growth factors, morphogens, and extracellular-matrix (ECM) proteins to shape the alveolar structure.^5^ When inflammation or oxidative stress disrupts this balance, alveolarization is stunted—resulting in fewer, larger alveoli and impaired capillary network formation.^7^

Historically, “**old BPD**” was characterized by fibrosis and inflammation secondary to high-pressure ventilation and excessive oxygen exposure. In contrast, “**new BPD**,” emerging under gentler ventilation and surfactant therapy, shows less fibrosis but persistent developmental arrest and architectural simplification.^6^ Abnormal AEC2 proliferation and differentiation remain central to this phenotype, marking these cells as both targets and mediators of injury.^4^

The ECM is no longer viewed as a passive scaffold but as a dynamic regulator of cell fate. Shifts in ECM composition during injury and repair alter integrin-mediated signaling and epithelial plasticity.^5, 10^ Within this dynamic microenvironment, matricellular proteins—especially CCN5—emerge as critical mediators of epithelial–mesenchymal communication and repair.^8^

## 3. Alveolar Epithelial Cell Biology and Plasticity

The alveolar epithelium comprises two principal cell types:

- **AEC1s:** thin, squamous cells covering more than 90% of the alveolar surface and enabling gas exchange.
- **AEC2s:** cuboidal cells producing surfactant and serving as progenitors that regenerate AEC1s after injury.

Following epithelial damage, AEC2s proliferate and differentiate into AEC1s to restore barrier integrity—a process governed by canonical signaling pathways such as Wnt/**β**-catenin, Notch, and Hippo/YAP.^10^

Therapeutic hyperoxia, a common intervention in neonatal intensive-care settings, disrupts AEC2 homeostasis by inducing oxidative stress and mitochondrial dysfunction.^7,9^ Hyperoxia leads to mitochondrial fragmentation, accumulation of reactive oxygen species (ROS), and premature senescence, all of which impair regenerative potential.

In addition, **epithelial-to-mesenchymal transition (EMT)**—a reversible program enabling epithelial cells to acquire mesenchymal characteristics—becomes exaggerated during chronic injury. While transient EMT contributes to wound repair, sustained activation promotes fibrosis.^4^ EMT is orchestrated by transcription factors such as *Snail*, *Slug*, and *Twist*, acting downstream of TGF-**β** signaling and ECM remodeling.^5^

Autophagy and metabolic reprogramming further shape AEC2 fate under stress. Impaired autophagy is associated with defective epithelial repair and increased apoptosis in neonatal hyperoxia models.^10, 9^

## 4. The CCN Family: Matricellular Mediators in Lung Biology

The CCN family consists of six secreted regulatory proteins—CCN1 through CCN6—originally recognized for their roles in growth-factor signaling and extracellular-matrix (ECM) modulation.^11^ Each protein contains four conserved structural domains: the insulin-like growth factor–binding protein (IGFBP) domain, von Willebrand factor type C repeat (VWC), thrombospondin type 1 repeat (TSP1), and cysteine-knot (CT) domain. Notably, **CCN5** lacks the CT domain, a distinction that contributes to its unique, largely anti-fibrotic properties.^8^

In pulmonary biology, **CCN2 (CTGF)** is the most studied member. It promotes fibroblast activation, collagen synthesis, and ECM deposition in idiopathic pulmonary fibrosis (IPF).^8^ **CCN1 (CYR61)**, in contrast, enhances angiogenesis and repair but may also potentiate inflammation under certain pathological conditions.^14^

**CCN5 (WISP2)** functions differently from its family counterparts. It inhibits fibroblast and smooth-muscle proliferation and counteracts TGF-**β**–induced profibrotic signaling. Evidence indicates that CCN5 stabilizes epithelial identity and suppresses epithelial–mesenchymal transition (EMT)—a hallmark distinguishing it from other CCN proteins.^1_3, 17^

## 5. Experimental Findings: CCN5 as a Protective Modulator

Our laboratory’s vestigations demonstrate that CCN5 expression is dynamically regulated in neonatal mouse lungs exposed to hyperoxia.^3^ Using a model of 85% oxygen exposure from postnatal day 0 to day 14, we observed:

- A significant **upregulation of CCN5 mRNA and protein** by days 3–7.
- **Localization of CCN5** predominantly in alveolar type II epithelial cells (AEC2s), with weaker expression in mesenchymal compartments.

**Functional studies** of adenoviral CCN5 overexpression in AEC2s revealed several protective effects:

- Enhanced cell viability during oxidative stress.
- Downregulation of mesenchymal markers (vimentin, **α**-smooth muscle actin).
- Suppression of EMT-associated transcription factors (*Snail1*, *Twist1*).
- Preservation of E-cadherin expression and tight-junction integrity.^1^

Conversely, **siRNA-mediated CCN5 knockdown** promoted apoptosis and EMT, confirming CCN5’s essential role in maintaining epithelial phenotype and barrier stability.^1^ In **epithelial–fibroblast co-culture mod**els, CCN5-overexpressing AEC2s attenuated fibroblast activation and collagen expression, suggesting a paracrine regulatory mechanism.^2^

Transcriptomic profiling of CCN5-overexpressing AEC2s under oxidative stress revealed **inhibition of TGF-β signaling** and enrichment of genes associated with antioxidant defense (e.g., *SOD2*, *GPX3*).^1, 3^ Collectively, these findings identify CCN5 as a central mediator of epithelial resilience and anti-fibrotic regulation in neonatal lung injury.

## 6. Cross-Talk Between Epithelium and ECM: CCN5 at the Intersection

Epithelial–stromal communication is fundamental to lung homeostasis and repair. The extracellular matrix (ECM) acts as a dynamic signaling interface, transmitting mechanical and biochemical cues through receptors such as integrins and syndecans. These signals activate downstream pathways including PI3K/AKT, YAP/TAZ, and MAPK, which collectively regulate epithelial proliferation, survival, and differentiation.^10^

**CCN5** appears to influence this crosstalk at multiple levels:

- It modulates integrin **α**v**β**6 and **α**6**β**4 interactions, which are key regulators of latent TGF-**β** activation.^4^
- It may bind directly to ECM components such as fibronectin and laminin, modifying their bioavailability and signaling potential.^5^
- By preserving epithelial polarity and tight-junction integrity, CCN5 helps prevent aberrant ECM remodeling and fibroblast activation that often follow epithelial barrier loss.^1^

Because fibrosis and alveolar arrest in bronchopulmonary dysplasia (BPD) are driven primarily by epithelial dysfunction and maladaptive ECM responses, targeting CCN5 to restore epithelial integrity offers a novel, mechanism-based therapeutic strategy.^8^

## 7. Therapeutic Opportunities and Regenerative Approaches Involving CCN5

The recognition of CCN5 as a protective, anti-fibrotic modulator in neonatal lung injury introduces several promising therapeutic possibilities.

### 7.1. GENE OR RNA DELIVERY

Viral vectors (adenoviral, adeno-associated, or lentiviral) and lipid nanoparticle systems could be used to enhance CCN5 expression in injured neonatal lungs. Because CCN5 is a secreted protein, localized expression has the potential to produce widespread paracrine effects on both epithelial and mesenchymal compartments.^8^

### 7.2. RECOMBINANT PROTEIN THERAPY

Recombinant CCN5 protein, delivered intranasally or via aerosol, may provide a noninvasive approach to replenish CCN5 in preterm infants at risk of BPD. Advances in protein engineering could stabilize CCN5 and optimize its receptor interactions for therapeutic use.^8^

### 7.3. SMALL-MOLECULE MODULATORS

Although no known compounds directly upregulate CCN5 expression, several pathways—including Wnt/**β**-catenin and estrogen signaling—indirectly enhance CCN5 transcription and activity.^12^ Pharmacologic agents targeting these upstream regulators could augment endogenous CCN5 as part of combination therapy for neonatal lung repair.

### 7.4. SYNERGISTIC THERAPIES

CCN5-based therapies could act synergistically with existing protective interventions, including:

- **Antioxidants** such as N-acetylcysteine (NAC) and vitamin A to reduce oxidative injury.^7^
- **Stem cell–derived extracellular vesicles (EVs)** from mesenchymal stem cells to promote epithelial recovery and reduce inflammation.^10^
- **Lung-protective ventilation protocols** to minimize barotrauma and mechanical stress.^6^

Together, these strategies highlight CCN5 as both a molecular target and a biomarker of epithelial restoration and redox homeostasis in neonatal lung disease.

## 8. Integration With Emerging Research

Independent investigations continue to underscore the critical importance of epithelial preservation in bronchopulmonary dysplasia (BPD) and related neonatal lung pathologies. Activation of YAP/TAZ signaling pathways has been shown to restore AEC2 function in hyperoxia models, emphasizing the regenerative potential of mechanical and molecular cues.^10^ Single-cell transcriptomic analyses reveal distinct AEC2 subpopulations marked by oxidative-stress gene signatures, correlating with impaired alveolarization and repair.^9^ Moreover, aberrant expression of ECM proteins such as periostin and tenascin-C disrupts epithelial stability, reinforcing the concept that matrix composition dictates epithelial fate.^5^

Clinically, biomarkers including E-cadherin fragments and matrix metalloproteinase-9 (MMP-9) detected in tracheal aspirates correlate with injury severity and repair outcomes.^7^ Given CCN5’s detectable presence in bronchoalveolar lavage and serum in experimental models, future studies should explore its utility as a predictive or diagnostic biomarker for neonatal lung injury.^3^

## 9. Knowledge Gaps and Future Directions

Despite strong preclinical knowledge, several questions remain unanswered:

- What upstream developmental or injury-induced signals regulate **CCN5** expression in neonatal lungs?
- Which specific receptors or binding partners mediate CCN5 activity in AEC2s?
- What are the long-term consequences of modulating CCN5 expression during early life?
- Does CCN5 overexpression influence susceptibility to later respiratory diseases such as asthma or COPD?
- Could CCN5 quantification serve as a biomarker for lung maturity or repair potential?

Addressing these gaps will require integrative approaches combining transcriptomics, proteomics, epigenomics, and in vivo functional modeling to delineate CCN5’s role within the broader regenerative network of the neonatal lung.^19^

## 10. Conclusion

Bronchopulmonary dysplasia remains a leading cause of long-term morbidity in survivors of extreme prematurity. Advances in developmental and molecular lung biology increasingly implicate epithelial–ECM signaling as a pivotal determinant of injury and repair outcomes. Among matricellular mediators, **CCN5** stands out as a uniquely protective factor—preserving epithelial identity, limiting mesenchymal activation, and stabilizing mitochondrial and redox homeostasis.^1_3, 8, 20^ Translating these insights into clinical interventions could transform the management of neonatal lung injury, providing strategies that promote true structural and functional regeneration rather than merely survival.
